# Responses to government-imposed restrictions: The sound of Australia's church bells one year after the onset of COVID-19a)

**DOI:** 10.1121/10.0006451

**Published:** 2021-10-12

**Authors:** Murray Parker, Dirk H. R. Spennenmann

**Affiliations:** Institute for Land, Water, and Society, Charles Sturt University, P.O. Box 789, Albury NSW 2640, Australia

## Abstract

The COVID-19 pandemic has demonstrated how a stochastic disruptive event can dramatically alter community soundscapes. Whilst religious bells have symbolism in many worldwide faiths, the sound emanating from church bells can be considered public domain and therefore, is not exclusive to the church. Pandemic-related interruption of these sounds impacts not only the church involved, but both the surrounding soundscape and any members of the community who ascribe value to these sounds. This paper examines the soundscape of Christian churches in the states of New South Wales and Victoria, to give an Australian perspective one year after the declaration of the COVID-19 pandemic in March 2020. It provides an update of the situation in Australia, building on our previous work from August of that year. In doing so, it explores the activity of church tower bell ringing, and how this “non-essential” activity has been affected, both during and subsequent to the heavy community restrictions applied in Australia. The paper also explores what lengths bellringers have undertaken to be permitted to conduct such activities, such as the use of adaptive measures due to “social distancing”, and considers what implications this enforced silence has in similar soundscapes elsewhere in the world.

## I. INTRODUCTION

In 2018–2019, the authors undertook a study examining church bell ringing in the state of New South Wales, Australia, with research questions investigating the extent of church bell ringing still practiced, what factors may determine this differentiation, and what values and significances were attributed to the bell ringing sounds by the practitioners themselves. While the full data is reported elsewhere (Parker and Spennemann, 2020b; Parker and Spennemann, 2021), we found that a high proportion of Anglican, Roman Catholic, and Orthodox churches retained bells on church premises, especially in churches of a historic period. For these churches that had bells, a large proportion of these denominations actually rang them, and there was subsequently a high level of perceived value placed on bell ringing, especially because it was considered a form of heritage. We then continued this research interest, using the COVID-19 pandemic as a case example of how a stochastic event can change one aspect of the sound world.

In August 2020, the authors investigated the effects of COVID-19 on a sub-set of the initial cohort, with specific interests in how bell ringing sounds had changed over six months in NSW (Parker and Spennemann, 2020a). This time frame included three periods of interest: previous to the first COVID-19 lockdown of April 2020, during this lockdown, and the subsequent post-lockdown period. We found that bells were largely silenced due to COVID-19, with ceased bell types including angelus bells, tolling bells, and pealing bells; angelus and tolling bells being a single individual bell, and pealing bells being a set of many bells (often 8) rung by a group of bellringers. Whilst some churches “snapped back” to pre-lockdown patterns of bell ringing, some churches did not return to these levels, and interestingly, some churches increased the capacity of bell ringing over the lockdown period. One year after the onset of COVID-19, noticing that Australia had multiple periods of lockdowns to varying extents and recognizing the potential issues of sound change in an urban setting, we chose to undertake a follow-up study to fully investigate patterns of church bell ringing change on both a larger scale and time frame.

## II. DATA SOURCES/METHODS

While the previous study looked at both single bells (tolling bells) and tower bells, we decided to follow up with a study in 2021 solely pertaining to tower bells, due to readily available data sources. The Australian and New Zealand Association of Bellringers (ANZAB) is a long-running (50 y) community entity promoting the art of change ringing, which maintains a complete register of towers containing ringing bells in these two countries. With the onset of the COVID-19 pandemic, they promptly set up a section of their website discussing which towers would be open and which towers would be closed due to differing governmental-imposed restrictions in the listed towers. To confirm data quality, individual contact with tower captains or other relative personnel was then made in cases of suspected errors on the website, as was the use of other public data, such as web pages and social media accounts. Data were collected on average bi-monthly, with follow up and verification via direct communication at the time of manuscript development. This information produced an accurate record showing when each individual tower opened up, over the period March 2020 to April 2021. We limited our study to the two most populous states in Australia—New South Wales and Victoria—with NSW having 16 towers in the capital city of Sydney and another 16 in regional towns, and Victoria having five towers in the capital city of Melbourne and another five towers in regional areas of this state.

## III. RESULTS

Over the entire annual period from late March 2020 to April 2021, we found that tower bell ringing in both NSW and Victorian churches was highly correlated with governmental-imposed restrictions which stipulated actions allowable by the community. Previously to any COVID impacts or restrictions, all of the bell towers in these two states listed by ANZAB were open and ringing as per normal. The results for NSW and Victoria (both metropolitan and regional) had different patterns, which can be attributed to the different restrictions imposed in either state.

**FIG. 1.**
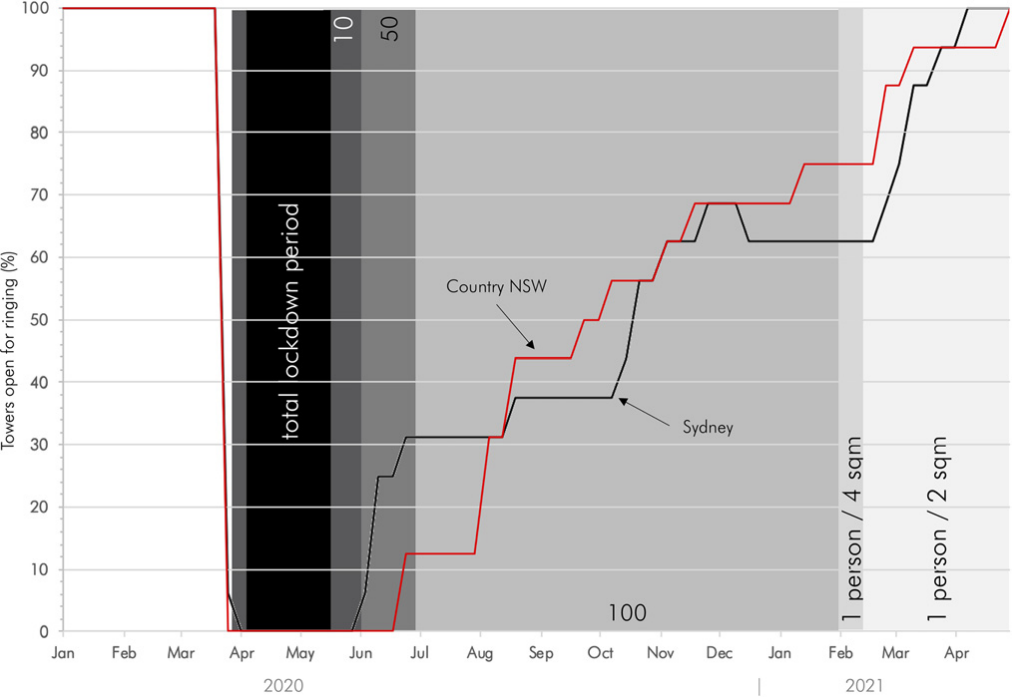
(Color online) The impact of COVID restrictions on the operations of church towers in New South Wales, January 2020–May 2021.

In NSW, the first case of community transmission of COVID was on March 2, 2020, with the state subsequently having a total lockdown period of about six weeks (Storen and Corrigan, 2020). During that time, no pealing bells were rung, and ringing was limited to single angelus/tolling bells at the decree of the diocese or parish (see Parker and Spennemann, 2020a). After the initial lockdown, social limitations changed—it was permitted to have ten people in a religious setting or space (i.e., church) in late May (MHMR, 2020a), and subsequently, 50 people in June (MHMR, 2020b). In Sydney over that period of time, there was an increase in church belltower ringing (30% of churches) through June and July. From July through February the cap on restrictions was lifted from 50 to 100 people, and there was reportedly another correspondingly large increase in church bell tower ringing over that period of time, to around 60% of pre-COVID levels. Restrictions were again subsequently lifted further to a capacity limit of one person per 4 m^2^, and finally, to one person per 2 m^2^ in April 2021. There was a much larger increase in church bell tower ringing over this time in the churches of Sydney, with levels of bell ringing approaching 100% (when these new restrictions were implemented) and hitting the full capacity of pre-COVID levels by the week of April 14, 2021. Regional churches showed a similar pattern of decline and return to bell ringing, although the lag times were greater than in metropolitan areas: the return to 30% capacity did not eventuate until August, and the final full capacity did not eventuate until April 21, 2021 (Figure 1).

**FIG. 2.**
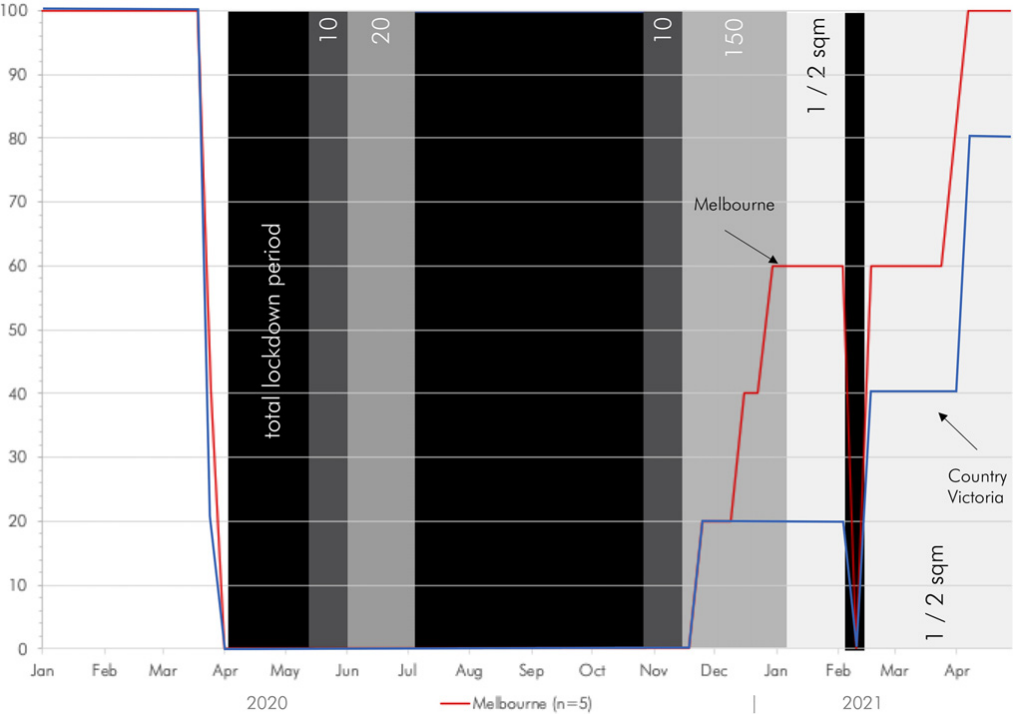
(Color online) The impact of COVID restrictions on the operations of church towers in Victoria, January 2020–May 2021.

Churches in Victoria showed a different decline and return pattern from those in NSW, which reflected restrictions pertinent to this individual state. It must be noted that the Victorian dataset is more limited. Having been founded 70 y after NSW, it is quite clearly reflected in the number of bell towers in the state; only five towers in Melbourne (capital city) and five towers in regional areas, compared to the 16 each in NSW. The Victorian shutdown began largely in the same way to NSW, with the first community transmission on March 7, followed by an initial gradual lockdown, and then a snap nationwide lockdown for six weeks (Storen and Corrigan, 2020). Similar to NSW, there was a gradual return of capacity in churches to ten and then 20 people (rather than 50), but unfortunately, community transmission reoccurred in Victoria and the state went again into a total lockdown (Melbourne in particular) until late October, with restrictions gradually lifting after that (Fig. 2).

During this entire period from April to mid-November 2020, there was again no tower bell ringing in this state. After November, a gradual easing of restrictions occurred, permitting 150 people in a congregation, followed by a one person per 2 m^2^ capacity rule in January 2021. There was an immediate positive response in bell ringing in Melbourne with a return to 60% of pre-COVID levels. In early February 2021, a case of community transmission caused a one-week snap lockdown across the state, and unlike the previous lockdowns of a gradual reduction of restrictions, this lockdown was sharp enough to allow a rapid bounce back to pre-levels, both in capacity limits and the prevalence of tower bell ringing. Full return of bell ringing to pre-COVID levels occurred by early April 2021 in Melbourne. Churches in regional Victoria showed a similar lag pattern, with only 20% (one of five bells) returning to ringing activities in the Christmas 2020 period, and only to 80% by the Easter period 2021 (Fig. 2).

## IV. DISCUSSION

**FIG. 3.**
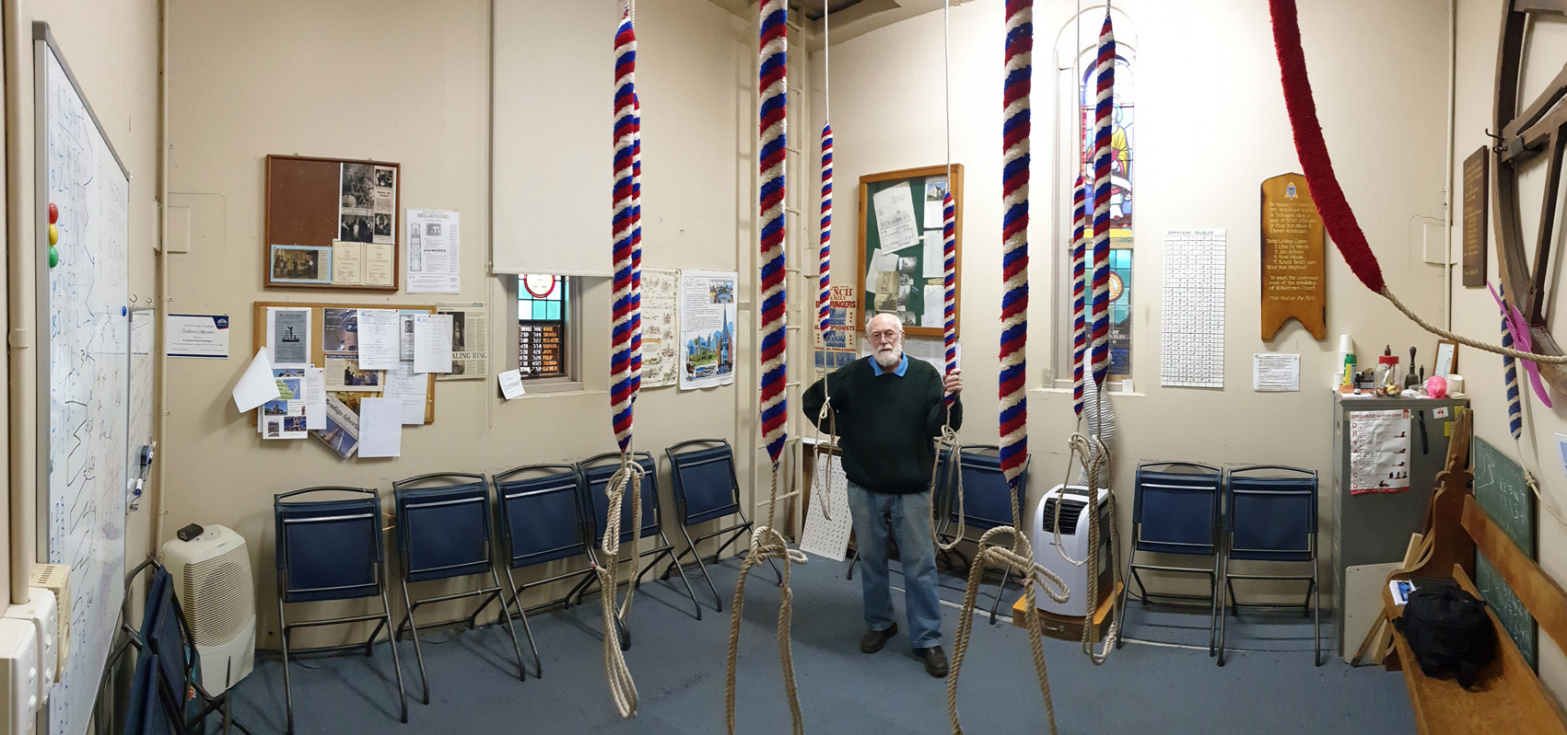
(Color online) The ringing room of St Matthews Anglican Church, Albury. Reprinted with permission from DHR Spennemann, 2020.

These delays in re-opening towers for bell ringing were not as expected at the commencement of the study. The driving force behind these delays across both regions of NSW and Victoria was the limitations imposed by the legally mandated “social distancing” between people in public spaces. Non-residential internal spaces were restricted to a maximum occupation density of one person per 4 m^2^ with further stipulation that persons other than members of the same household had to socially distance at 1.5 m (around 5 ft) apart (MHMR, 2020c). In a standard ringing room containing eight ropes, the spacing between the bell ringers is fairly close in a normal operation procedure, with bell ropes placed around 3 ft apart (Fig. 3). Such close distance between bellringers, however, is not permitted during tight restrictions. In order to allow eight people the distance of 1.5 m apart (the legal requirement), the bellringers would essentially have their backs against the walls. While that may be potentially possible, the setup and length of the ropes and the rhythmic motion required for the bell ringing activity do not make this a viable option in most cases. Whilst some towers, such as St Mary's Cathedral, Sydney, were able to resume soon after the first lockdown (ringing from June 7 and practice sessions from July 2), this was primarily due to the large amount of space in the ringing room, alongside strict protocols in place of distancing, and hand hygiene (Australian and New Zealand Association of Bellringers, 2020). Other church towers would have smaller spaces to contend with. As such, any visible lag from the dataset is largely a result of the various bell ringing communities working out options for making the tower work, given the space and safety constraints they had during this period of time.

During the return to regular bell ringing, we discovered some really interesting adaptive responses being employed in some churches, both for bell training and in public bell ringing. In order to continue some form of training for the bellringing team, some tower groups advocated for the use of “virtual ringing rooms” during the height of the pandemic, using web-based applications where the actual ringing action is undertaken by pressing a key to ring an individual bell. It was noted that while it kept mathematical functions working cognitively, it offered none of the actual physicality of ringing (Goodin, 2020). One church, St James' (Queen's Square), Sydney, allowed a small number of the regular band of ringers to return to the tower for weekly practice using a simulator, as social constraints could allow the two people needed in the bell chamber for this activity— one to ring and another to operate the simulator (Dettman, 2021).

**FIG. 4.**
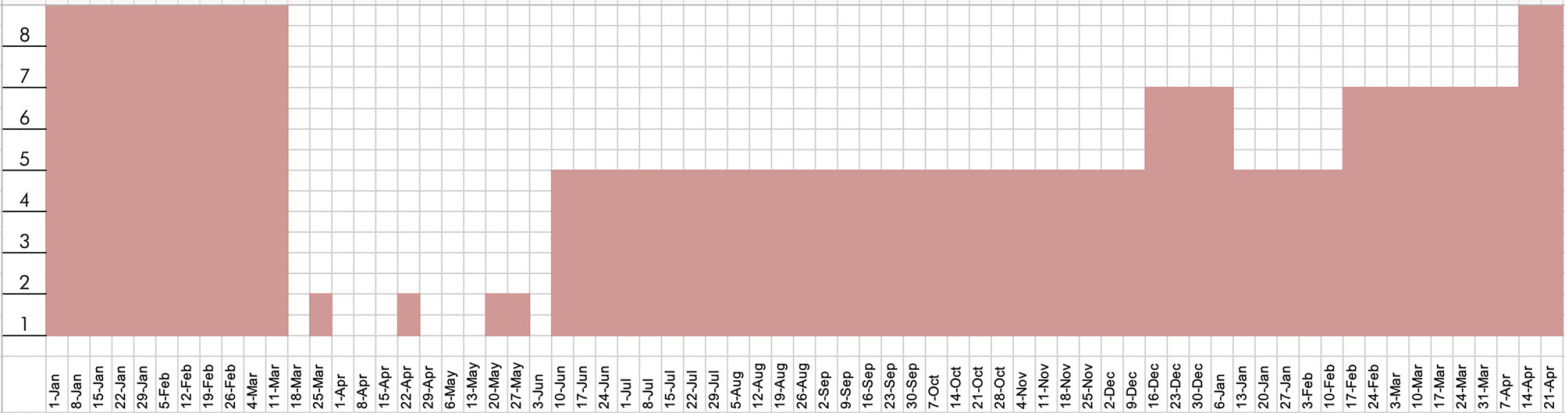
(Color online) Number of tower bells rung at St. Paul's Anglican Church (Burwood NSW) from January 2020–April 2021.

Other churches applied adaptive measures when actually ringing tower bells, in an attempt to return to the activity level pre-COVID, while still complying with restrictions. For example, at St Paul's Anglican Church, Burwood (NSW), eight bells were rung in the period pre-COVID, which was the total maximum, as it is an eight-bell tower (Fig. 4). During the height of the lockdown, no bells were calling except for one single tenor bell which tolled on special occasions and for outdoor church services on March 22, 2020 and March 29, 2020, and again from May 17 (Brock, 2020). Once the restrictions lifted somewhat, the church returned to half capacity (four bells) from June 7 for much of the remainder of 2020, then increased to six bells during the Christmas period, decreased back down to four with higher restrictions, increased to six on February 14, 2021 and finally, to eight bells on April 12, 2021 (Brock, 2021a; 2021b). Despite having the capacity for eight bells in the tower, the church was not able to ring at this capacity for most of the year due to the imposed restrictions. Similar measures were undertaken by ringers at St. Jude's Randwick, Sydney. After months of no ringing, activity was resumed and restricted to four bells with appropriate social distancing. By early 2021, it was common to have six, seven, or all eight bells ringing on Sunday morning services at this church (Langford-Brown, 2021).

This presents an interesting case for discussion: the ringing of tower bells during the COVID-19 pandemic is therefore not so much a question of the presence or absence of bells, but more a question of the richness of the sound that is able to be rung due to the number of available bells as a result of space restrictions. The reason follows thus: Ringing a certain number of bells allows specific repertoire to be performed; eight bells allows the performance of Basingstoke Surprise Major, and six bells allow Grandsire Doubles. However, limiting the number of bells actually restricts the repertoire available to be performed, and instead of scalic patterns, the church has to offer other suggestions, such as triadic patterns or similar. These restrictions, therefore, do not only create limitations on the number of churches ringing bells, but a change in the manner of richness in the soundscape of the surrounding area. Further adaptive measures allowed some churches to conquer this, such as the ringing of non-adjacent bells—there were cases of churches ringing Basingstoke with four bellringers, with bellringers having two bells each; and other towers utilized family groups for ringing purposes as the restrictions in this situation were not as strict. We discovered similar instances of adaption. For example, Plain Bob was documented being performed having only three bellringers using two bells each, and Oxford Treble Bob Minor using only three bells with three ringers, rather than the usual six bells.

Furthermore, our research uncovered other aspects of change pertaining to the soundscapes created by church bell ringing, including differences in duration and intensity of emanated sound. Following government health advice recommending limiting the time spent within an indoor space, bell ringing duration was reduced from 1.5–1 h at weekly practice sessions at All Saints Church, Singleton (NSW), and limited to 15 min solely for services at Hoskins Uniting Church, Lithgow (NSW) under a specially devised COVID-19 Safety Plan (Musgrave, 2020; Cox, 2021). Of particular interest were the adaptive measures undertaken at St James' Old Cathedral, Melbourne, whereby regular ringing on Sundays was enacted using an Ellacombe apparatus, a system that allows the clappers to be pulled against the bells by just one individual, but resulted in a volume which was significantly lower than usual (Goodin, 2021). Whilst outside the scope of this paper, further research utilizing acoustic measurements could be undertaken to investigate sound pressure levels and spectral variations of these altered sound environments.

At the time of writing (September 2021), it is important to note that parts of Australia have again returned to a total shutdown of non-essential activities due to increased cases of community transmission of the COVID-19 virus. Greater Sydney has been shut down for at least eleven weeks and regional NSW for three weeks, Melbourne for five weeks, and regional Victoria for two weeks. It is expected that an enforced lockdown will continue for some time into the future, and with it, a continued hiatus of tower bell ringing across both metropolitan and regional areas of NSW and Victoria. However, it is important to note that the cessation of church bell ringing is in no way limited to states of Australia. As of April 2021, one year from the onset of COVID-19, it was reported that there had only been a total of five tower bell peals rung throughout the world, and all of these had been rung in NSW (the term “peal” relates to a performance of change ringing meeting particular conditions for duration, complexity, and quality), and of the 99 tower bell quarter peals rung worldwide, 68 had been rung in towers associated with the Australian and New Zealand Association of Bellringers (ANZAB) (Peard, 2021). Indeed, there was a total worldwide hiatus on any open peal bell ringing from March 20, 2020 (St. Clement's Yass NSW: 5040 Surprise Minor) until October 5, 2020 (St Mary's Cathedral, Sydney NSW: 5000 Spliced Royal). The issue of a changed soundscape with reference to church bell ringing and restriction associated silence is therefore a global issue, affecting any countries that both ring church bells and have been impacted by the pandemic.

## V. CONCLUSION

Clearly, COVID-19 had quite a dramatic impact on the practice of bellringers in both NSW and Victoria and the way in which they could practice their art. It basically silenced the towers for the entire initial lockdown period and then subsequent periods. Even if towers reopened due to capacity restriction changes, restrictions limited the number of people being allowed to engage and participate in the bell ringing activity, so any regular practicing activity had been effectively ceased. Whilst gradual return allowed the recommencement of the practicing activity, we must keep in mind that as the art form of bell ringing requires excellent timing, missing almost one year's worth of physical practice could negatively impact any art community. We show in this example that COVID-19 had a potentially massive impact on community wide soundscapes, first in the silencing of bell sounds; and in cases where sounds were effectively permitted, such as the ringing of an individual tolling bell, the rich sound created by tower bells was basically silenced. While we know these sounds did gradually return, at the moment we do not know what the impact of this change in soundscape had on the community well-being. Whilst we can report and discuss statistics whether churches did or did not ring, social science studies have not instigated research looking at the effect of lack of sound on people. We also need to recognize that we currently do not have any pre-COVID measurements of community well-being scales either. This presents opportunity for future research, and these are activities that we should investigate in the current climate, as it would be prudent to assume that the COVID-19 pandemic will not be the last pandemic to affect our society.
